# Supplementary material for: Mapping Research Trends in Frailty and Nutrition: A Combined Bibliometric and Structured Review (2000–2024)
Source: Nutrients. 2025 Nov 12;17(22):3541. doi: 10.3390/nu17223541 (PMC12655092; doi:10.3390/nu17223541)
Supplement: Supplementary file 1 [file nutrients-17-03541-s001.zip › nutrients-3928702-supplementary.pdf]

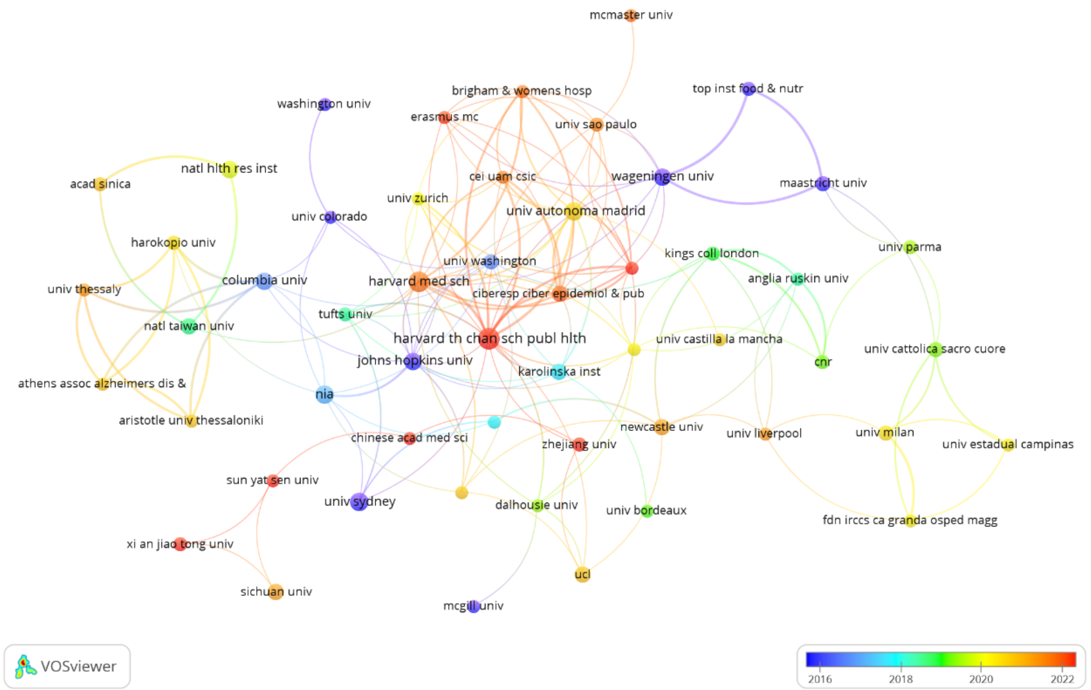

Figure S1. Institutional Collaboration Network

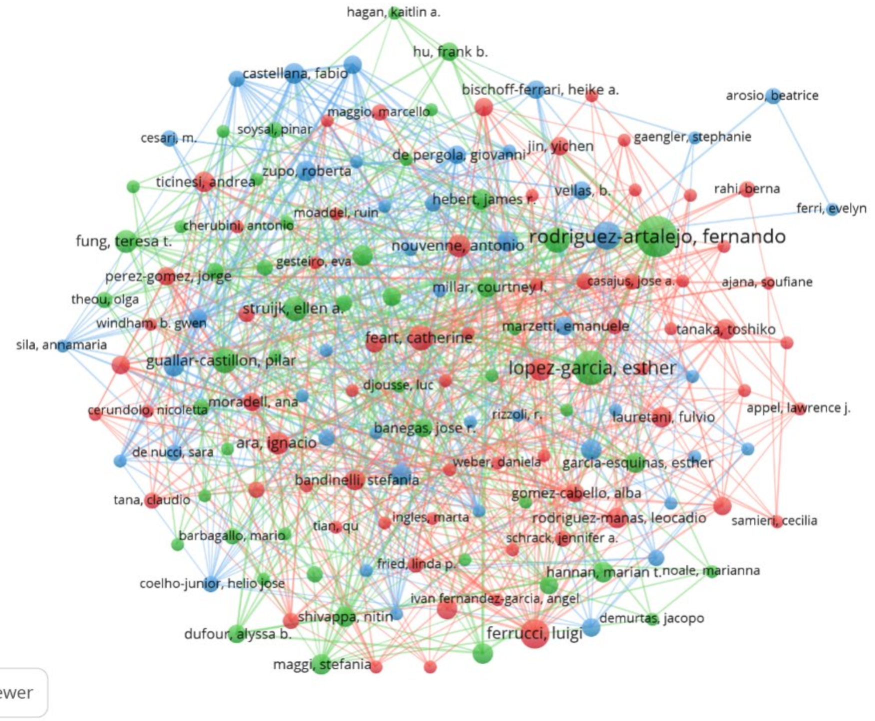

Figure S2. Author Collaboration Network

Table S1. Top 10 co-cited references

Rank	Co-cited Reference	Citations
1	Fried LP, 2001, j gerontol a-biol, v56, pm146	452
2	Clegg A, 2013, lancet, v381, p752	119
3	Morley JE, 2013, j am med dir assoc, v14, p392	96
4	Lorenzo-lópez L, 2017, bmc geriatr, v17	94
5	Bartali B, 2006, j gerontol a-biol, v61, p589	89
6	Collard RM, 2012, j am geriatr soc, v60, p1487	74
7	Folstein MF, 1975, j psychiat res, v12, p189	74
8	Beasley JM, 2010, j am geriatr soc, v58, p1063	71
9	Hoogendijk EO, 2019, lancet, v394, p1365	71
10	Rockwood K, 2005, can med assoc j, v173, p489	70

Table S2. 15 highly cited reference contents

Rank	Strength	Main research content
1	15.39	Frailty in elderly people
2	15.1	Nutritional determinants of frailty in older adults: A systematic review
3	14.07	Frailty Consensus: A Call to Action
4	13.92	Macronutrients, Diet Quality, and Frailty in Older Men
5	10.95	Dietary Quality Is Related to Frailty in Community-Dwelling Older Adults
6	10.5	Major dietary patterns and risk of frailty in older adults: a prospective cohort study
7	10.41	A Higher Adherence to a Mediterranean-Style Diet Is Inversely Associated with the Development of Frailty in Community-Dwelling Elderly Men and Women
8	9.46	Dietary Patterns and Risk of Frailty in Chinese Community-Dwelling Older People in Hong Kong: A Prospective Cohort Study
9	9.02	Evidence-Based Recommendations for Optimal Dietary Protein Intake in Older People: A Position Paper From the PROT-AGE Study Group
10	8.97	Preventive Effect of Protein-Energy Supplementation on the Functional Decline of Frail Older Adults With Low Socioeconomic Status: A Community-Based Randomized Controlled Study
11	8.66	Frailty: implications for clinical practice and public health
12	8.44	Mediterranean Diet and Risk of Frailty in Community-Dwelling Older Adults
13	8.44	Frailty and nutrition: Searching for evidence
14	8.15	Protein Intake and Incident Frailty in the Women's Health Initiative Observational Study
15	8.7	Management of frailty: opportunities, challenges, and future directions

Table S3. Top 20 high-frequency keywords

Rank	Keywords	Counts
1	frailty	870
2	nutrition	237
3	older adults	168
4	aging	171
5	sarcopenia	159
6	elderly	143
7	malnutrition	139
8	nutritional status	103
9	vitamin D	85
10	diet	107
11	exercise	75
12	inflammation	67
13	older people	50
14	frailty index	46
15	mediterranean diet	57
16	frail elderly	36
17	gut microbiota	43
18	protein	55
19	aged	53
20	mortality	36

Table S4. Studies categorization

By study design	Counts
Cross-sectional studies	127
Cohort studies	95
Clinical trials	35
By nutritional category	Counts
Micronutrients	62
Macronutrients	51
Food groups	37
Dietary patterns	84
Nutritional interventions	23
